# Gestational Weight Gain Intervention Impacts Determinants of Healthy Eating and Exercise in Overweight/Obese Pregnant Women

**DOI:** 10.1155/2018/6469170

**Published:** 2018-10-01

**Authors:** Abigail M. Pauley, Emily Hohman, Jennifer S. Savage, Daniel E. Rivera, Penghong Guo, Krista S. Leonard, Danielle Symons Downs

**Affiliations:** ^1^Exercise Psychology Laboratory, Department of Kinesiology, The Pennsylvania State University, University Park, State College, PA, USA; ^2^Center for Childhood Obesity Research, Department of Nutritional Sciences, College of Health and Human Development, The Pennsylvania State University, University Park, PA, USA; ^3^School for Engineering of Matter, Transport, Energy, Arizona State University, Tempe, AZ, USA; ^4^Department of Obstetrics and Gynecology, College of Medicine, The Pennsylvania State University, Hershey, PA, USA

## Abstract

High gestational weight gain (GWG) in overweight/obese pregnant women increases maternal-fetal complications. We conducted a 6-week GWG intervention based on an energy balance model that includes theories of planned behavior (TPB) and self-regulation constructs to promote exercise and healthy eating motivation and behaviors. The purposes of this proof-of-concept feasibility study were to examine: (1) the energy balance model constructs over the intervention, and (2) pre-post intervention, weekly, and dose-response changes in study constructs. *Methods*. Overweight/obese pregnant women (*N*=17) were randomized to 1 of 6 conditions, increasing in intensity, and included varied combinations of components (exercise sessions, healthy eating demonstrations, etc.). Exercise and healthy eating TPB (attitude, subjective norm, perceived behavioral control, intention), and self-regulation (prospective, retrospective) constructs were collected weekly. Exercise behavior, energy intake, and GWG were collected daily. *Results*. We observed: (a) significant increases in exercise TPB constructs, healthy eating attitude (limit unhealthy foods), exercise/healthy eating retrospective self-regulation; (b) significant decrease in healthy eating subjective norm (limit unhealthy foods); (c) trending increases for healthy eating perceived behavioral control (limit unhealthy foods), healthy eating prospective self-regulation, and energy intake; (d) significantly higher active time, steps, and energy expenditure at W3 relative to other weeks; (e) no significant increase in GWG; and, (f) a dose response effect such that women in more intensive dosages had greater gains in exercise and healthy eating perceived behavioral control (eat healthy/limit unhealthy foods). *Conclusion*. Brief exposure to a theoretically-driven, GWG intervention resulted in changes to exercise and healthy eating TPB and self-regulation motivational determinants, no significant increase in GWG, and suggests intervention intensity can strengthen perceived ability to engage in exercise/healthy eating behaviors; offering initial proof-of-concept for the intervention to regulate GWG in overweight/obese pregnant women. Future research will test this intervention over the course of pregnancy to understand long-term impact on maternal-fetal health outcomes.

## 1. Introduction

Over half of all overweight and obese pregnant women gain weight in excess of the current gestational weight gain (GWG) recommendations [1, 2]. This is problematic because high GWG increases the risk for preterm delivery, gestational diabetes, vascular disease, hypertensive disorders of pregnancy, and postpartum weight retention [1]. Even more alarming, high GWG elevates the risk for fetal morbidity including macrosomia (i.e., birth weight >4,000 g regardless of gestational age), birth trauma (e.g., shoulder dystocia), and longer hospital stays [1, 3]. Data from the National Vital Statistics System [2] indicate that a high percentage of overweight (61%) and obese (55%) women are at greater risk for exceeding the IOM GWG guidelines (i.e., total weight gain: 15–25 pounds for overweight; 11–20 pounds for obese) and therefore warrant intervention to effectively manage GWG for optimal maternal and fetal health.

Of particular concern, the rate of overweight/obese pregnant women exceeding GWG guidelines is predicted to increase given the lack of a “gold standard” intervention or clinical treatment [4]. Furthermore, the majority of interventions that aimed to reduce GWG in overweight/obese pregnant women have either yielded minimal effects or have been unsuccessful altogether [5–9]. However, information can be learned from the few effective studies. Vesco et al. [10] conducted a group-based weight management intervention in obese pregnant women using an energy-reduced DASH diet, recommendation of 30 minutes of moderate physical activity, and individual and group education sessions. They observed lower GWG in the intervention group from baseline (7–21 weeks gestation) through follow-up (34 weeks gestation) compared to the control group. Sagedal et al. [11] conducted an intervention where pregnant women received dietary counseling and twice-weekly exercise classes. They observed lower mean GWG in the intervention group compared to controls who received routine prenatal care. Despite these findings, the majority of participants in both studies still exceeded the IOM GWG recommendations. Sagedal et al. [11] also reported the proportion of women who exceeded the IOM GWG guidelines did not differ between condition, suggesting that a more intensive intervention approach (e.g., individually tailored exercise and dietary guidance) addressing the unique needs of overweight/obese women is needed to effectively manage GWG [4, 11, 12].

Given this premise, we developed an individually tailored GWG intervention to manage weight in overweight/obese pregnant women based on a model of energy balance that includes the theories of planned behavior (TPB) and self-regulation [13–19]. The TPB aims to explain behavior through underlying constructs of attitude (positive or negative evaluation of the behavior), subjective norm (perceived social support to engage in the behavior), and perceived behavioral control (ease or difficulty in performing the behavior), which influence intention (person's level of motivation) to perform or not perform a behavior such as exercise or healthy eating [20]. Self-regulation is the ability for a person to work towards a goal by monitoring and managing thoughts, feelings, and behaviors, which is critical to changing behaviors such as exercise and healthy eating [21, 22]. Targeting both the TPB constructs and self-regulation simultaneously within an intervention can strengthen the likelihood of changing behavior, especially when the intervention is individually tailored based on the participant's level of the construct, for example, using strategies such as daily self-monitoring, positive encouragement, and reinforcement for a woman with low perceived behavioral control for exercising and eating healthy.

Previous work by Symons Downs et al. [23] established the utility to increase exercise determinants in pregnant women with gestational diabetes, but there is little research that has examined the associations between TPB and self-regulation constructs among pregnant women within the context of a GWG management intervention, especially in overweight/obese pregnant women. Thus, we conducted a brief, 6-week trial of an intervention focusing on exercise and healthy eating motivation and behaviors to manage GWG in overweight/obese pregnant women [18, 19]. The overall goal of this proof-of-concept trial was to establish feasibility of the intervention components and the extent to which the TPB and self-regulation constructs motivated overweight/obese pregnant women to engage in exercise and healthy eating behaviors (as exercise and healthy eating are essential for regulating GWG). The purposes of this feasibility study were to (1) descriptively examine the energy balance model constructs over the 6-week pilot intervention and (2) examine pre-post intervention, weekly, and dose-response changes in the study constructs. Since the intervention period was brief, and intervention behavior change often takes longer than 6 weeks, we anticipated to observe changes in the underlying TPB and self-regulation motivational determinants without our energy balance model rather than observing changes in behavior (exercise, healthy eating, and GWG) [24]. We also expected to observe a dose-response effect such that the TPB and self-regulation constructs would improve with an increase in intervention dosage intensity. Our future research will test this GWG intervention in a larger trial over the course of pregnancy to understand long-term changes in exercise, healthy eating, and GWG as well as impact on additional maternal-infant outcomes.

## 2. Methods

### 2.1. Participants

**Figure 1:**
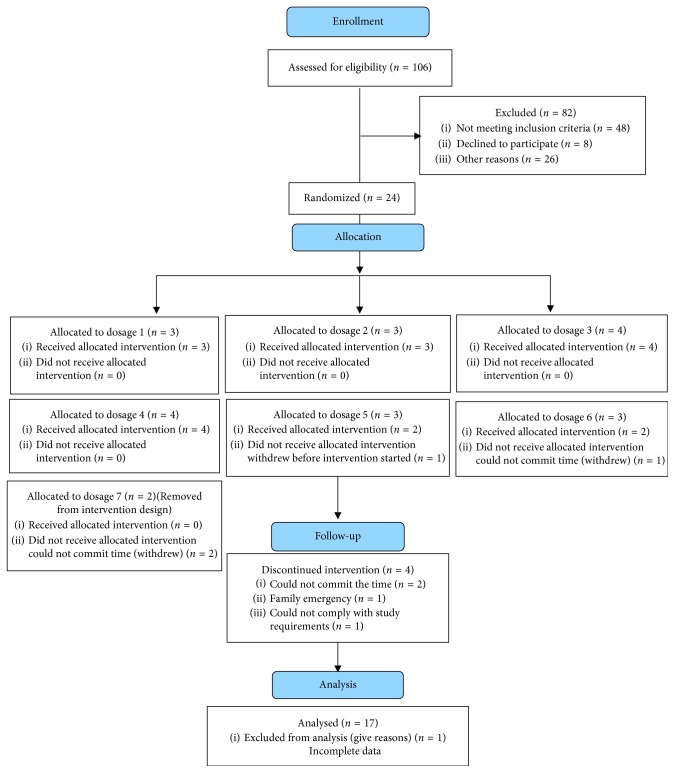
Consort recruitment table.

Overweight/obese pregnant women (*N*=17; *M* age = 29.4 years, SD = ± 5.6) were recruited using on-site clinic (e.g., speaking with potential participants after a prenatal appointment) and community (e.g., ads, flyers, and word of mouth) methods in locations in Central Pennsylvania. Women were randomized to 1 of 6 intervention dosages, which increased in intensity, for 6 weeks using a statistician-developed scheme placing an equal distribution of overweight and obese women in each dosage (Figure 1). Inclusion criteria were first pregnancy (no prior full-term (≥37 weeks gestation) births), between 12 and 28 weeks gestation, 18–45 years old, body mass index (BMI) ≥ 25, English-speaking, and no contraindications to exercise or eating healthy foods [25, 26]. Physician's consent was obtained from the women's obstetrics and gynecology providers prior to enrollment.

### 2.2. Study Design

#### 2.2.1. Baseline and Follow-Up Assessments

Participants were met at the University's Clinical Research Center, explained study procedures, and obtained informed consent. Eligible and interested women completed a brief medical exam to ensure safety for participation followed by measures of their height, weight, and blood pressure. During the baseline assessment, women also completed self-reported measures of exercise and healthy eating TPB (attitude, subjective norm, perceived behavioral control, and intention) and self-regulation (prospective and retrospective) constructs and personal demographics (e.g., age and BMI). Women were given instructions on how to complete self-reported measures using an online data capture system (REDCap; [27]). Women were given a wrist-worn exercise activity monitor (to measure active time, energy expenditure in kcal, and steps), Wi-Fi weight scale, and instructions on how to use these devices as well as how to complete diet records using a smartphone app for three days during baseline. During the intervention, women completed weekly measures of the exercise and healthy eating TPB and self-regulation constructs and daily measures exercise behavior (wrist-worn activity monitor) and weight (Wi-Fi scale); energy intake was assessed three days/week using the smartphone app for dietary record intake, but for the current analyses, a back-calculation method was used for energy intake (described below). The same procedures (not including the medical exam and technology trainings) were used to collect data at the follow-up assessment.

#### 2.2.2. Intervention

**Figure 2:**
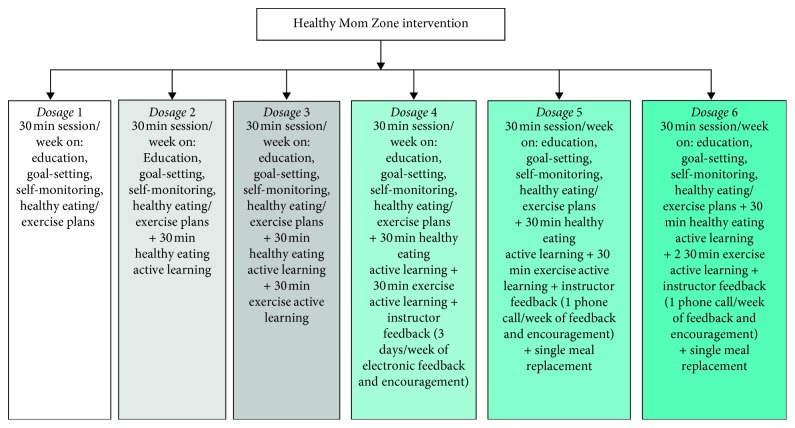
Healthy mom zone intervention dosages description.

Intervention dosages were based on principles of behavior change, TPB, and self-regulation [20, 28, 29], content from past successful interventions [23, 30], and initial prototype development feedback from pregnant women. Intervention dosages were built upon one another in a “step-up” design for intensity such that Dosage 1 included the “baseline intervention” with education on principles of exercise, healthy eating, GWG, goal-setting, and self-monitoring. Dosage 2 included the baseline intervention (i.e., Dosage 1) + a “step-up” of 30 min of healthy eating active learning (e.g., cooking demonstrations, recipe preparation, understanding principles of portion size, and energy density). The dosages increased in intensity with added exercise sessions, self-monitoring with instructor feedback, and healthy eating meal replacements up through Dosage 6. We originally designed the intervention with seven dosages; however, feedback from women during prototype development found this dosage to be too intensive, so we removed it from the study design.  Figure 2 illustrates the six intervention dosages that were tested in this study.

### 2.3. Measures

Study measures of exercise and healthy eating TPB and self-regulation were collected weekly for six weeks (i.e., week 1 = baseline, weeks 2–5 of intervention, and week 6 = follow-up) via online surveys. Intensive longitudinal data inform interventions and efficacy by capturing people's lives while they are actually living [31] which is a different approach from conventional experimental/survey research. All TPB constructs were measured on a 7-point Likert scale with higher scores indicating more positive attitude, subjective norm, perceived behavioral control, and intention.

#### 2.3.1. Exercise TPB Constructs

*Attitude* was assessed with 7 differential pairs (e.g., useless-useful) that described how women felt about exercising for at least 30 minutes/day of accumulated moderate physical activity on most, if not all, days of the week [32, 33]. *Subjective Norm* was assessed with three items (e.g., strongly disagree-strongly agree) measuring perceived support from important others to exercise for 30 minutes/day on most days in the following week. *Perceived Behavioral Control* was assessed with 3 items (e.g., for me to be physically active each day in the next week will be: extremely difficult/very little control/strongly disagree-extremely easy/complete control/strongly agree). *Intention* to engage in 30 minutes of exercise on most days of the week was assessed with six items (e.g., I intend to be physically active each day in the next week: strongly disagree-strongly agree). Internal consistency scores for the exercise TPB measures ranged from alpha = 0.77–0.98 from pre-post for all constructs. Compliance of the exercise TPB measures was 99%.

#### 2.3.2. Healthy Eating TPB Constructs

Authors developed the healthy eating TPB scale by modifying the validated exercise TPB items. *Eating Attitudes* were assessed with 14 differential pairs, 7 assessing healthy eating attitudes (how women felt about eating healthy foods each day in the next week) and 7 assessing attitudes about limiting unhealthy foods (i.e., sugary beverages, eating chips, candy, baked goods, and fried foods each day in the next week). *Subjective Norm* was assessed using six items; three items measured women's perceptions of the extent to which significant others (e.g., husband, mother, and friend) provided support for them to eat healthy each day in the next week and three items assessed perceived support from others to limit unhealthy foods. *Perceived Behavioral Control* was assessed using six items; three items measured the ease or difficulty in eating healthy foods each day in the next week and three items assessed the ease or difficulty in limiting unhealthy foods each day in the next week. *Intention* was assessed using 12 items (e.g., strongly disagree/definitely not/not at all-strongly agree/definitely/very much). Three items were adapted to healthy eating from the exercise intention measure. Three items asked about women's intention to limit unhealthy foods. Three items assessed women's intention/motivation/plan to eat healthy and three items assessed intention to limit unhealthy foods each day in the next week. Internal consistency scores for the healthy eating and limiting unhealthy food TPB measures ranged from alpha = 0.73–0.98 from pre-post for all constructs. Compliance of both, the healthy eating and limiting unhealthy food TPB measures, was 99%.

#### 2.3.3. Exercise Self-Regulation

Exercise self-regulation was assessed with 16 items to evaluate the degree to which women self-regulated their exercise behavior (e.g., if I achieve my short-term (daily or weekly) goals for exercise, I will feel proud; [28, 29, 34]). Eight items assessed prospective (i.e., in the next week) and eight items assessed retrospective (i.e., in the past week) self-regulation. Items were divided into 7 subscales: self-monitoring, goal-setting, action planning, coping planning, scheduling, cuing, and affective reaction. Internal consistency scores were excellent (alpha = 0.92–0.97) across time points. Compliance of the exercise self-regulation measure was 99%.

#### 2.3.4. Healthy Eating Self-Regulation

Healthy eating self-regulation was assessed with 16 items to evaluate the degree to which women self-regulates their healthy eating behavior (e.g., if I achieve my short-term (daily or weekly) goals for eating healthy, I will feel proud; [28, 29]). Eight items assessed prospective and eight items assessed retrospective healthy eating self-regulation. Items were divided into 7 subscales: self-monitoring, goal-setting, action planning, coping planning, scheduling, cuing, and affective reaction. Internal consistency scores ranged from alpha = 0.83–0.93 across the time points. Compliance of the healthy eating self-regulation measure was 99%.

#### 2.3.5. Exercise Behavior

Exercise behavior was assessed using a wrist-worn activity monitor to measure daily activity time, energy expenditure, and steps [35, 36]. Women wore the activity monitor 24 hours/day over the entire intervention period. Each woman had her own wrist monitor that connected to her phone via a mobile app to use as a self-monitoring tool. Compliance with the monitors was 93%.

#### 2.3.6. Energy Intake

Energy intake was assessed using a back-calculation equation of energy intake [16] to address concerns about under/overreporting of energy intake when using self-report (pencil and paper or smartphone app) food records [37]. In the calculation, the participant's weight and energy expenditure are used to predict energy intake. Energy intake was predicted for each day over the 6-week intervention using average kcal/day.

#### 2.3.7. GWG

GWG was assessed daily using a Wi-Fi scale over the 6-week study period. Women weighed themselves each day as soon as they woke up. The scale transmitted weights automatically to secure participant online accounts; online data were accessed and stored in REDCap. Compliance with the scale was 87%.

### 2.4. Data Analysis

Descriptive statistics were used to examine study means, standard deviations, and frequencies. Changes in TPB and self-regulation scores from baseline to follow-up were calculated with *t*-tests. Repeated-measures ANCOVA controlling for gestational age, dosage (1–6), and prepregnancy BMI was conducted to examine the weekly changes in the planned behavior/self-regulation constructs, exercise, energy intake, and GWG. Effects of study dosage on pre-post changes were assessed with ANOVA. Analyses were performed in SAS 9.4 (SAS Institute, Cary, NC) and SPSS 25 (IBM Corp., Armonk, New York). As this is a pilot study, the *p* value for statistically significant differences overtime was set to *p* ≤ 0.05 and the *p* value for trends overtime was set to *p* ≤ 0.10 [38].

## 3. Results

### 3.1. Demographics

The sample (*N*=17) was homogenous; most participants were Caucasian, married, completed college, and had a family income of $40,000 or higher per year (Table 1). Prepregnancy BMI of participants was in the overweight range (*M* BMI = 29.1 kg/m^2^, SD = ± 3.8, range = 24.7–39; 65% overweight, 35% obese). Women were randomized to 1 of 6 dosages: Dosage 1 *n*=3, Dosage 2 *n*=3, Dosage 3 *n*=4, Dosage 4 *n*=4, Dosage 5 *n*=2, and Dosage 6 *n*=2.

### 3.2. Pre-Post Intervention Change in Energy Balance Model Constructs

**Figure 3:**
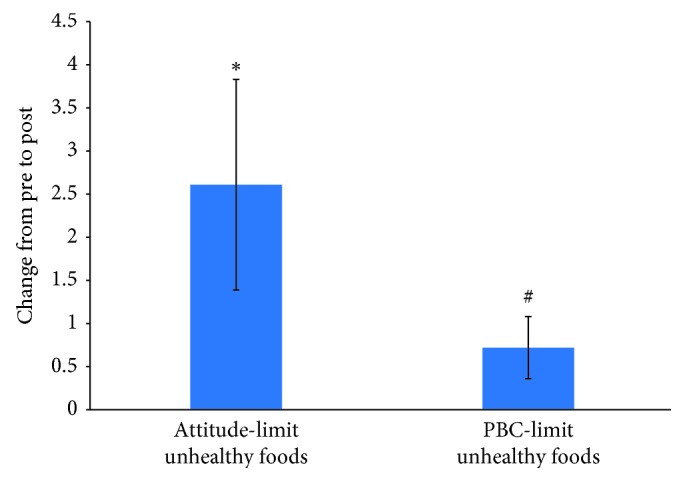
Change in TPB limit unhealthy eating constructs from pre- to post-assessment. Values are mean ± SE, ^*∗*^*p* < 0.05, ^#^*p* < 0.10.

There was a significant increase in healthy eating attitude (limit unhealthy foods, *p*=0.046) and a trend for an increase in healthy eating perceived behavioral control (limit unhealthy foods, *p*=0.06, Figure 3). There was a significant increase in retrospective exercise and healthy eating self-regulation (*p*=0.004, *p*=0.0001). There were no significant changes for any of the other exercise/healthy eating TPB or self-regulation constructs, exercise and healthy eating behaviors, or GWG.

### 3.3. Weekly Change in Energy Balance Model Constructs

#### 3.3.1. Exercise TPB

The repeated-measures model for exercise attitude was significant, Wilks lambda = 0.258, *F*=4.613, *p*=0.028. Overall, attitude increased from W1–W6 of the intervention with significant differences at W2–W4 (*p*=0.044) and trending differences at W2–W6 (*p*=0.078). The repeated-measures model for exercise subjective norm was also significant, Wilks lambda = 0.271, *F*=4.301, *p*=0.034. Subjective norm increased from W1–W6; however, these differences did not reach statistical significance. The repeated-measures models for exercise perceived behavioral control and intention were not significant, Wilks lambda = 0.900, *F*=0.178, *p*=0.963, and Wilks lambda = 0.537, *F*=1.379, *p*=0.326, respectively. However, exercise perceived behavioral control and intention had significant differences at W2–W5 (*p*=0.014, *p*=0.017, respectively); perceived behavioral control also had trending differences at W2–W6 (*p*=0.070; Table 2).

#### 3.3.2. Healthy Eating TPB

The repeated-measures model for healthy eating attitude, subjective norm, perceived behavioral control, and intention was not significant, Wilks lambda = 0.487, *F*=1.687, *p*=0.243, Wilks lambda = 0.728, *F*=0.523, *p*=0.753, Wilks lambda = 0.786, *F*=0.435, *p*=0.813, and Wilks lambda = 0.388, *F*=2.522, *p*=0.118, respectively. There were no significant changes across weeks. The repeated-measures models for healthy eating attitude, perceived behavioral control, and intention (limit unhealthy foods) were not significant, Wilks lambda = 0.493, *F*=1.646, *p*=0.253, Wilks lambda = 0.553, *F*=0.969, *p*=0.503, and Wilks lambda = 0.631, *F*=0.934, *p*=0.507, respectively. There were no significant changes across weeks. The repeated-measures model for healthy eating subjective norm (limit unhealthy foods) was significant, Wilks lambda = 0.094, *F*=9.659, *p*=0.013. Subjective norm decreased from W1–W6; however, these differences did not reach statistical significance (Table 2).

#### 3.3.3. Exercise and Healthy Eating Self-Regulation

The repeated-measures model for exercise self-regulation prospective was not significant, Wilks lambda = 0.744, *F*=0.549, *p*=0.736; there were no significant differences across weeks. The repeated-measures model for exercise self-regulation retrospective was also not significant, Wilks lambda = 0.566, *F*=1.227, *p*=0.379. However, significant weekly increases were observed from W2–W4, W2–W5, and W2–W6 (*p*=0.028, 0.023, 0.043, respectively). The repeated-measures model for healthy eating self-regulation prospective trended toward significance, Wilks lambda = 0.358, *F*=2.873, *p*=0.089. Healthy eating self-regulation prospective increased from W1–W6; however, these differences were not statistically significant. The repeated-measures model for healthy eating self-regulation retrospective was not significant, Wilks lambda = 0.542, *F*=1.350, *p*=0.335. However, significant week increases were observed from W1–W3, W1–W4, W1–W6, and W2–W4 (*p*=0.017, 0.014, 0.016, 0.043, respectively) and a trending increase was observed at W1–W5 (*p*=0.079) (Table 3).

#### 3.3.4. Exercise and Healthy Eating Behavior

The repeated-measures model for active time was not significant, Wilks lambda = 0.494, *F*=1.024, *p*=0.490; however, there were significant differences at W2-W3, W3-W4, and W3–W6 (*p*=0.007, *p* < 0.001, *p* < 0.001, respectively) and trending differences at W3–W5 (*p*=0.086) such that W3 had significantly higher active time (e.g., 96 min) compared to the other weeks (e.g., 72 min on average). The repeated-measures models for step and active kcal were also not significant, Wilks lambda = 0.877, *F*=0.169, *p*=0.965, and Wilks lambda = 0.841, *F*=0.189, *p*=0.954, respectively. However, steps and active kcal at W3 were significantly higher than at W6 (*p*=0.013, *p*=0.006, respectively). The repeated-measures model for energy intake trended toward significance, Wilks lambda = 0.003, *F*=73.617, *p*=0.088. Overall, energy intake increased from W1–W6 of the intervention by 197 kcal; however, these weekly differences were not statistically significant (Table 4).

#### 3.3.5. GWG

The repeated-measures model for GWG was not significant, Wilks lambda = 0.521, *F*=1.150, *p*=0.430, and there were no significant changes across weeks (Table 5). GWG did not significantly increase over the 6-week intervention; observation of mean GWG across the weeks showed a similar pattern of approximately 1.1 pound change/week over the study period.

### 3.4. Dose-Response Change in Energy Balance Model Constructs

**Figure 4:**
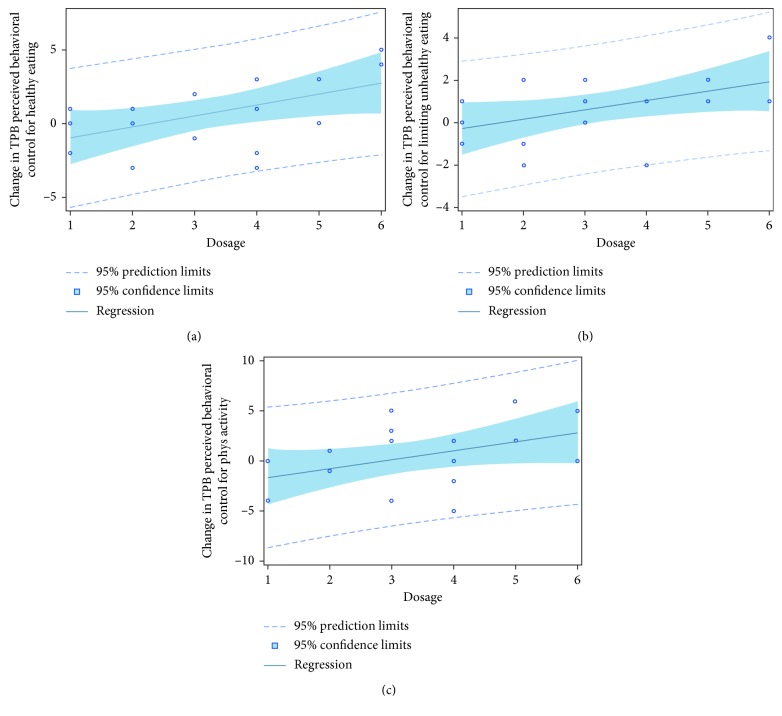
Intervention dose was positively associated with pre-post change in perceived behavioral control for (a) healthy eating (*p*=0.03), (b) limiting unhealthy eating (*p*=0.03), and (c) exercise (*p*=0.07).

There was a positive relationship between study dosage and pre-post change in exercise perceived behavioral control (*p*=0.07; Figure 4(c)), healthy eating perceived behavioral control (*p*=0.03; Figure 4(a)), healthy eating perceived behavioral control for limiting unhealthy foods (*p*=0.03; Figure 4(b)), healthy eating intention (*p*=0.04), and healthy eating intention for limiting unhealthy foods (*p*=0.03). In other words, women randomized to receive dosages at higher intensities had greater intention to eat healthy/limit unhealthy foods and their perception of eating healthy/limiting unhealthy foods and exercising was more positive. There were no significant dose-response pre-post changes in exercise and healthy eating self-regulation, exercise and healthy eating behaviors, or GWG.

## 4. Discussion

The objectives of this feasibility study were to descriptively examine the energy balance model constructs over the 6-week pilot intervention and examine pre-post intervention, weekly, and dose-response change in the study constructs. Overall, we found that brief exposure to the theoretically driven, GWG intervention resulted in significant changes to some of the exercise and healthy eating TPB and self-regulation motivational determinants, an increase in exercise behaviors at W3 of the intervention, and no significant changes to energy intake or GWG, supporting the initial proof-of-concept of the intervention among overweight/obese pregnant women. We also observed a dose-response effect such that an increase in intervention dosage was associated with greater exercise and healthy eating perceived control. These findings are further discussed below.

In partial support of our assumption, the intervention resulted in changes to some of the TPB and self-regulation motivational determinants over the intervention period. More specifically, we observed significant increases in exercise attitude, subjective norm, perceived behavioral control, and intention, healthy eating attitude (limit unhealthy foods) and exercise/healthy eating retrospective self-regulation, a trend toward significance for an increase in healthy eating perceived behavioral control (limit unhealthy foods) and healthy eating prospective self-regulation. These findings are consistent with our past research among pregnant women with gestational diabetes [23], and they suggest that brief exposure to the intervention can positively impact how women feel about exercise and healthy eating.

These findings also illustrate a positive impact on women's perceived ability and self-regulation for exercise and healthy eating, which in turn can have a positive impact on these behaviors to help better regulate GWG. More specifically, the intervention education and skills (including exercise and healthy eating action plans) taught to the women along with the use of mHealth tools (e.g., Wi-Fi scale, wrist-worn activity monitor, and smartphone app for intake served as both intervention self-regulatory tools and behavioral measures) positively influenced women's retrospective and prospective self-regulation. These findings also suggest that women felt more comfortable with regulating their exercise and eating behaviors from week to week and were able to think about setting goals and action plans in advance of an upcoming week. These findings are promising for the future intervention that will require women to self-regulate exercise and healthy eating behaviors over the duration of pregnancy to regulate their weight gain. While we did not find significant pre-post intervention change in exercise perceived behavioral control and intention, there were significant increases in exercise perceived behavioral control and intention from W2–W5 and a trend toward significance for exercise perceived behavioral control from W2–W6. This suggests that the accumulation of the education and skills learned over the course of the short intervention period provided the women with tools to manage perceived barriers and improve their intention for exercise. These weekly findings are promising for the design of the future intervention as they may be replicated over a longer intervention period. Similarly, with a trend toward significance for perceived behavioral control (limit unhealthy foods), the program content appears to provide women with useful strategies to overcome key barriers to eating healthy foods and improved their perceived ability to reduce consumption of unhealthy foods. The positive findings for increases in exercise perceived behavioral control and healthy eating perceived behavioral control (limit unhealthy foods) are particularly important for designing interventions as perceived behavioral control is a strong determinant of behavior in pregnant women [32] and will play a key role in the future intervention to manage GWG over the entire course of pregnancy.

Moreover, while we found increases in exercise subjective norm and healthy eating attitude (limit unhealthy foods), we interestingly observed a significant decrease in healthy eating subjective norm (limit unhealthy foods) over the intervention period. One explanation for these findings may be that women are exposed to intervention content on strategies to exercise and eat healthy, and this content partly focuses on the role that friends and family have in supporting these behaviors. While the women themselves may have improved their own feelings about the benefits of exercise and eating healthy through the intervention exposure, it is possible that some of the women also gained insight to barriers in their support system to eating healthy (e.g., husband or family poor eating habits) that decreased their subjective norm for limiting unhealthy foods. Future research is needed to better understand these normative influences for limiting unhealthy foods; additional intervention content on social influences may be needed in the future larger intervention to help women overcome these barriers.

Although we did not assume that brief exposure of the intervention would result in significant changes to exercise and healthy eating behaviors, we nevertheless observed some interesting findings. For example, although exercise activity time at W6 of the intervention was slightly lower (71 min) compared to W1 (75 min), exercise activity time was considerably higher at W3 (96 min) compared to all other weeks (e.g., range 65–75 min), and there was a significant increase in time from W2-W3. A similar pattern was observed for activity steps and energy expenditure kcal with the highest observed values at W3 of the intervention. It is possible that women started to feel more comfortable with the intervention by W3, and this resulted in a spike in their activity, but then they may have encountered some barriers (e.g., soreness or fatigue from exercise; thinking they were doing “too much too fast”) that led to a decline in activity following W3. Even though the exercise sessions recommend activity within the guidelines (150 min/week of moderate intensity aerobic activity [25, 26]), the recommendation may have been perceived to be more intense by some than it actually was. Given that intervention exposure longer than six weeks is generally needed to see impact on behavioral changes [24], and we did not obtain weekly feedback on the women regarding specific exercise barriers, some caution is warranted with interpreting these findings. However, considering these findings in combination with the promising evidence for the positive effects of the intervention on the exercise motivational determinants, there is proof-of-concept for the intervention impacting exercise behaviors. Future research is needed to test this assumption over a longer period of time during pregnancy to understand changes in exercise as a result of the intervention.

Moreover, while energy intake trended toward a significant increase over the study period, it is important to note that (a) pregnant women are supposed to increase their energy intake in pregnancy and adjust it relative to their activity level [39], and (b) the observed increase from W1 to W6 was only a difference of approximately 200 kcal. In other words, there is proof-of-concept for the intervention to regulate energy intake such that it did not lead to a significant increase in kcal over the study period. Also, caloric needs for pregnant women differ across the trimesters, so the increase in kcal could be related to the range in gestational age of the participants [39] (e.g., gestational age at study start ranged from 12 to 25 weeks and at study end, ranged from 18 to 31 weeks). Increases in caloric needs over the course of pregnancy need to be taken into consideration when developing interventions to manage GWG. Future research is needed to understand the impact of our intervention on energy intake over the entire pregnancy and in relation to GWG. Furthermore, although we did not anticipate to find any significant differences in GWG, it is nevertheless important to note that we did not observe a significant increase in GWG over the course of the intervention period. The average change in GWG from week to week was ∼1.1 pounds. Although this rate of change is higher than is recommended for overweight/obese pregnant women (e.g., average range of 0.4–0.7 pounds per week after the 1st trimester for overweight and obese, respectively; [1]), it is promising that we did not observe any large spikes in weight gain for most of the women. Future research is needed to test our intervention over the entire pregnancy to fully understand the impact on GWG.

Also, in partial support of our assumption, we observed a dose-response pre-post change in exercise perceived behavioral control, healthy eating perceived behavioral control, healthy eating perceived behavioral control (limit unhealthy foods), healthy eating intention, and healthy eating intention (limit unhealthy foods) but not exercise or healthy eating attitude or subjective norm. The dose-response change in exercise perceived behavioral control is important to highlight because with the higher dosage, the woman's perceived ease to exercise increases as they are provided with the tools to perform the behavior and know it is safe. With the feeling of increased ease to exercise, women are more likely to continue with exercise, which in turn will help manage energy balance for healthy weight gain throughout pregnancy. Similarly, the dose-response in perceived behavioral control healthy eating and healthy eating (limit unhealthy food) suggests that with a higher intervention dosage, there is greater perceived ease of eating healthy and limiting unhealthy foods. Higher intervention dosages included more intensive education materials, greater number of cooking demonstrations, and meal replacements, which provided women with the perceived resources and skills to make it easier to eat healthy and limit unhealthy foods. Also, in higher dosages, women had greater intention to eat healthy foods and to limit unhealthy foods. Coupled with the positive increase in attitude to limit unhealthy foods from pre- to post-intervention, and the trend for an increase in perceived behavioral control to limit unhealthy foods, these findings illustrate proof-of-concept for our intervention dosages. This is particularly important as our future intervention trial will be adapted over the entire pregnancy to help women adjust to their individual needs. For example, if a woman is successfully regulating her weight gain and staying within her GWG goals, she will continue with the baseline intervention (e.g., similar to Dosage 1 in this study). However, if another woman is struggling with regulating her GWG within her goals, the intervention will be adapted (i.e., made more intensive by increasing the dosage as illustrated in dosages 2–6 in this study) in 3- to 4-week cycles to manage GWG [19].

## 5. Implications for Practice and/or Policy

These study findings are important for clinical practice as they illustrate the importance of targeting motivational determinants for behavior change in addition to exercise and healthy eating to regulate GWG. Clinicians may want to consider talking with their overweight/obese prenatal patients more specifically about their attitudes, feelings, and barriers to making behavioral changes to better regulate their weight gain. Strengths of this study include the theoretically driven basis of the intervention, focus on overweight/obese women, population in need of managing GWG for healthy maternal and infant outcomes, randomized study design, and impact of the intervention on several outcomes after brief exposure. This study is also one of the first interventions to target both TPB and self-regulation constructs in an energy balance model for regulating GWG in overweight/obese pregnant women. These strengths are important for developing future interventions as well as disseminating information when helping pregnant women to regulate their GWG.

Limitations of this study include a small sample size with a homogenous sample (e.g., married, middle to upper class, Caucasian), which limits generalizability. Future research will aim to broaden generalizability of our study findings. Also, given the short duration of the intervention, we did not anticipate to see significant behavioral changes in exercise, energy intake, or GWG. Future research will need to examine the impact of the intervention on these behavioral targets over the full duration of pregnancy. Furthermore, although the healthy eating TPB measures were adapted from the validated exercise TPB measures, future research is needed to validate these items within our energy balance model.

## 6. Conclusions

Brief exposure to a theoretically driven, GWG intervention based on a model of energy balance resulted in significant increases to exercise and healthy eating motivational determinants, an increase in exercise in the middle of the intervention, and no significant changes to energy intake or GWG, supporting the initial proof-of-concept of the intervention among overweight/obese pregnant women. Future research will test this intervention in a larger trial over the entire duration of pregnancy to understand its impact on regulating GWG and maternal-infant health.

## Figures and Tables

**Table 1:** Demographic characteristics of the study sample.

Variable	Total sample *N*=17		
	M	SD	%
Age	29.4	5.6	
Body mass index (BMI)	29.1	3.8	
Gestational age at study start	16.4	4.5	
Weight at study start (pounds)	185.74	27.49	
Weight gain over 4-week dosage (pounds)	6.6	4.5	

*Marital status*			
Married			75.0
Not married living with partner			18.75
Single			6.25
Divorced			0

*Race*			
Caucasian			94.5
Hispanic			5.5

*Education*			
Graduate			23.5
College			76.5
High school			0

*Family income*			
$100,000			29.4
$40–$100,000			52.9
$20–$40,000			11.7
$10–$20,000			5.9

*M* = mean; SD = standard deviation.

**Table 2:** Exercise and healthy eating theory of planned behavior repeated measures.

	Week 1		Week 2		Week 3		Week 4		Week 5		Week 6		Wilks lambda	*F*	Partial eta squared	*p* value
	*M*	SD	*M*	SD	*M*	SD	*M*	SD	*M*	SD	*M*	SD				
TPB exercise attitude (*N*=16)	6.08	0.83	6.09	0.67	6.31	0.60	6.32	0.68	6.22	0.70	6.32	0.79	**0.258**	**4.613**	**0.742**	**0.028** ^*∗*^
TPB exercise subjective norm (*N*=16)	5.75	1.24	5.77	1.12	5.88	1.39	6.04	1.24	5.9	1.17	5.96	1.19	**0.271**	**4.301**	**0.729**	**0.034** ^*∗*^
TPB exercise perceived behavioral control (*N*=16)	5.27	1.04	4.29	0.89	5.35	1.04	5.17	1.16	5.56	0.98	5.38	0.97	0.900	0.178	0.100	0.963
TPB exercise intention (*N*=16)	5.41	1.39	5.23	0.97	5.58	1.00	5.33	1.1	5.67	0.99	5.44	1.21	0.537	1.379	0.463	0.326
TPB healthy eating attitude (*N*=16)	6.30	0.695	6.30	0.41	6.37	0.55	6.36	0.57	6.40	0.64	6.47	0.59	0.487	1.687	0.513	0.243
TPB healthy eating subjective norm (*N*=15)	6.29	0.93	6.18	1.10	6.04	1.11	6.16	1.17	6.02	1.23	6.04	1.27	0.728	0.523	0.272	0.753
TPB healthy eating perceived behavioral control (*N*=16)	5.75	0.68	6.79	0.75	5.85	0.69	5.81	1.2	5.92	0.63	6.02	0.75	0.786	0.435	0.214	0.813
TPB healthy eating intention (*N*=16)	5.92	0.98	6.05	0.74	6.07	0.83	5.93	0.87	6.01	0.86	6.13	0.93	0.388	2.522	0.612	0.118
TPB limit healthy eating attitude (*N*=16)	5.92	0.85	6.19	0.58	6.21	0.6	6.20	0.65	6.19	0.68	6.32	0.64	0.493	1.646	0.507	0.253
TPB limit healthy eating subjective norm (*N*=13)	6.41	0.81	6.23	0.92	6.05	1.10	6.15	1.18	6.10	0.81	6.00	1.34	**0.094**	**9.659**	**0.906**	**0.013** ^*∗*^
TPB limit healthy eating perceived behavioral control (*N*=14)	5.64	0.86	5.98	0.83	5.83	0.87	5.62	1.13	5.74	0.83	5.88	0.82	0.553	0.969	0.447	0.503
TPB limit healthy eating intention (*N*=16)	5.56	1.28	5.78	1.03	5.74	0.91	5.65	0.89	5.73	0.96	5.85	0.95	0.631	0.934	0.369	0.507

^*∗*^ *p* < 0.05; ^#^*p* < 0.10.

**Table 3:** Exercise and healthy eating self-regulation repeated measures.

	Week 1		Week 2		Week 3		Week 4		Week 5		Week 6		Wilks lambda	*F*	Partial eta squared	*p* value
	*M*	SD	*M*	SD	*M*	SD	*M*	SD	*M*	SD	*M*	SD				
Exercise prospective (*N*=16)	40.63	6.69	37.55	6.28	35.88	6.66	37.19	8.71	38.06	8.66	36.5	8.85	0.744	0.549	0.265	0.736
Exercise retrospective (*N*=16)	28.00	9.32	30.81	8.78	33.97	7.63	35.49	10.54	36.35	9.71	36.04	10.47	0.566	1.227	0.434	0.379
Healthy eating prospective (*N*=16)	39.33	8.05	36.56	6.23	36.19	7.19	38.21	7.15	37.85	8.51	38.38	8.1	**0.358**	**2.873**	**0.642**	**0.089** ^#^
Healthy eating retrospective (*N*=16)	25.87	9.70	31.25	9.45	34.03	7.13	36.63	8.24	34.56	9.83	37.13	9.25	0.542	1.350	0.458	0.335

^*∗*^ *p* < 0.05; ^*#*^*p* < 0.10.

**Table 4:** Exercise and healthy eating behavior repeated measures.

	Week 1		Week 2		Week 3		Week 4		Week 5		Week 6		Wilks lambda	*F*	Partial eta squared	*p* value
	*M*	SD	*M*	SD	*M*	SD	*M*	SD	*M*	SD	*M*	SD				
Active time (*N*=13)	75.05	23.1	71.27	27.76	96.38	34.60	73.95	31.16	65.68	30.27	71.72	29.04	0.494	1.024	0.506	0.490
Steps (*N*=14)	7674	2688	7917	3323	8582	2997	8235	3345	7205	3228	7042	2794	0.877	0.169	0.123	0.965
Energy expenditure (*N*=13)	467.2	202.2	447.4	236.5	507.4	218.3	497.9	251.3	405.3	234.4	411.7	212.8	0.841	0.189	0.159	0.954
Energy intake (*N*=9)	2755	928	2780	475	2935	541	2732	668	2887	550	2952	593	**0.003**	**73.62**	**0.997**	**0.088** ^#^

^*∗*^ *p* < 0.05; ^*#*^*p* < 0.10.

**Table 5:** Change in GWG repeated measures.

	ΔW1-W2		ΔW2-W3		ΔW3-W4		ΔW4-W5		ΔW5-W6		Wilks lambda	*F*	Partial eta squared	*p* value
	*M*	SD	*M*	SD	*M*	SD	*M*	SD	*M*	SD				
GWG (*N*=12)	1.50	1.17	0.65	1.48	1.08	0.94	1.00	0.87	1.59	0.85	0.521	1.150	0.479	0.430

^*∗*^ *p* < 0.05; ^*#*^*p* < 0.10. Weight is measured in pounds.
